# Neurological manifestation of coeliac disease with particular emphasis on gluten ataxia and immunological injury: a review article 

**Published:** 2021

**Authors:** Dina Osman, Seemeen Umar, Humayun Muhammad, Esmaeil Nikfekr, Kamran Rostami, Sauid Ishaq

**Affiliations:** 1 *North Cumbria University Hospital NHSF Trust, UK *; 2 *York Teaching Hospital, NHSF Trust, UK *; 3 *University of Roehampton, London, UK*; 4 *Nottingham University Hospitals, Queen's Medical Centre, UK*; 5 *Department of Gastroenterology Mid-Central District Health Board, Palmerston North, New Zealand*; 6 * Dudley Group of Hospitals NHSF Trust, UK*

**Keywords:** Coeliac disease, Gluten ataxia, Gluten enteropathy

## Abstract

Coeliac disease (CD) is a gluten-induced enteropathy affecting 1% of the population and has extra intestinal manifestations. One such expression involves nervous system, and CD may present as gluten ataxia (GA), peripheral neuropathy and epileptiform disorder among others. Considerable controversy exists on the exact pathophysiological mechanism of gluten leading to ataxia. It is, however, clear that in intestinal axis tissue transglutaminase 2 (tTG2) is the primary target but in the nervous system, tTG6 may be the causative antigen although its exact role is not clear. Furthermore, it has also been postulated that anti-gangliodise antibodies may play a role in the emergence of central pathology if not the key contender. Moreover, the association of neurological injury with non-coeliac gluten sensitivity (NCGS), a related but pathologically different condition implies an independent mechanism of neuronal injury by gluten in the absence of CD. This review will touch on the salient features of CD and the nervous system and will highlight current controversies in relation to gluten and GA.

## Introduction

 Coeliac disease (CD) is a T-cell-mediated chronic autoimmune disorder affecting 1% of the population (1), characterised by permanent intolerance to gluten (2) in genetically predisposed individuals (3). Histologically, CD is characterised by villous flattening and lymphocytosis of the small bowel mucosa (4) which may lead to malabsorption of micronutrients (5). This, in turn, generates variable clinical presentations based on the presence or absence of certain symptoms (6). When symptomatic, CD classically leads to diarrhoea, weight loss and abdominal pain (7). Moreover, CD affects multiple body organs (8) and apart from damage to the small bowel mucosa, it may also affect skin (9) bone (10), liver (11) thyroid (12), pancreas (13) and heart (14). In the majority of cases, the condition responds to a gluten-free diet (GFD) (15), only to relapse after the reintroduction of gluten into the diet (16,17). Additionally, CD may also lead to complications such as osteoporosis (18), anaemia (19) and possible intestinal lymphoma (20). 

Being a multi-system disorder (21, 22), CD may present as a range of abnormalities in the nervous system (23). This may occur at any time during the course of disease, i.e., either before or after the classical symptoms and signs of CD appear (24-26), and it may be the sole manifestation of the disease (25). Furthermore, on the neurological spectrum, it may manifest as cerebellar ataxia (27), peripheral neuropathy (28), seizures (29), headache (30), cognitive impartment (31,32) and various neuropsychiatrist disorders (33). Moreover, the nervous system is affected commonly as suggested by a recent prospective study indicating that up to 40% of newly diagnosed CD patients suffer from some form of neurological illness based on data from patients’ history, examination by an expert neurologist along with magnetic resonance (MR) spectroscopy and supporting serology. This notion was well supported by a recent detailed systematic review by Mearns et al., (34) who reported that 39% of patients suffered from gluten neuropathy in the diagnosed CD. 

Neurological manifestations are thought to occur in 6-10% of patients with cerebellar ataxia, the most frequent neurological involved area in CD (35, 36). This article will review the relationship of CD with Cerebellar Ataxia (CA) and immunopathology of enteric CD in comparison to neuronal pathology. The role of gluten-free diet (GFD) will also be reviewed with special emphasis on its effect on improving symptoms of ataxia. In this way, some controversies will be resolved in the context of ataxia associated with gluten. It is, however, accepted that the complexity of the topic arises from the unclear pathogenesis, prevalence in different population groups and the impact of gluten-free diet on neurological symptoms and signs. 

**Cerebellar Ataxia**

The term *ataxia *denotes a syndrome of imbalance and incoordination involving gait, limbs, and speech (37), and the underlying aetiology can be usually localized to the cerebellum where neural integration of information, coordination, and planning is impaired. Additionally, the spinal cord, vestibular system and peripheral sensory nerves may be affected which may impair proprioception, hence resulting in problems with balance and equilibrium (38). Ataxia can be broadly classified based on the aetiology into genetic or acquired/degenerative categories. Major causes of the latter include: Arnold-Chiari and Dandy Walker malformations, neoplasia related, medications, vascular, infections, inflammation and metabolic (39). Moreover, CD is a recognised cause of ataxia, first described in mid-sixties in a group of patients (n=16) with cerebellar atrophy and Purkinje Cell (PC) loss on post-mortem analysis (40) . 

The term gluten ataxia (GA) was developed about two decades ago (41,42). This is one of the commonest associations with CD (36) and is defined as sporadic ataxia associated with anti-gliadin antibodies, provided that other causes of ataxia are excluded (43), and which may affect up to 6% of patients with CD (34). Similarly, 40% of the patients with neurological deficit in CD have GA (44,45). Additionally, this is a relatively less disabling disease in terms of dependence on other for mobility (44). 

**Mechanism of neurological injury in gluten ataxia**

The exact mechanism of neurological injury in GA is not clear partly because neurological injury in CD *per se* is a complex issue arising from multiple mechanisms including nutritional deficiencies, immunological injury, toxic insults and metabolic mechanisms (24,31,46). Accordingly, immunologically mediated injury has been a well-established entity in nervous system (47-51) and ataxia, especially CA (47-49,52,53). To understand the immunopathology, it is important to examine it from both enteric and neurologic ends of the injury. 

From the intestinal end, there are relatively clear pathways of immunological injury induced by antigen-antibody interaction. It is postulated that in conjunction with HLA (DQ2/8) gluten (antigen) is presented to α/β receptor bearing pre-sensitised T lymphocytes (54,55) which, in turn, activate a series of events leading to the production of cytokines (56) by involving both humoral and cellular immunity (57-59) leading to mucosal pathology as described by Marsh and colleagues (60). Possible explanation may be related to the loss of tolerance and immunological response directed against primary autoantigen such as tissue transglutaminase (tTG) which is a family of nine related members in humans, involved in post-translational modification of proteins (61). 

In the small intestine, tTG is involved in enzymatic cross-linking reactions, merging amine, and deamination leading to cell adhesion, signal transduction and cellular apoptosis (61,62). It is established that tTG-2, a sub-member of the family, is the primary target in intestinal epithelium in gastrointestinal manifestation of CD (63,64). Similarly, tTG-3 seems to play a role in the manifestation of the skin condition, dramatis herpetiformis, associated with CD, (65) but considerable debate exists regarding what is the immuno-pathological mechanism for gluten injury in the CNS.

From the nervous system point of view, in pathological terms, spinal fluid has had paucity of findings (66) and histo-pathologically patchy changes are observed in the cerebellum, brain stem and spinal cord characterised by multi-focal neuronal loss (67). In patients with GA, a difference in neuronal physiology was identified as measured by MR spectroscopy in comparison to healthy controls, suggesting the objective role of inflammation in the cessation of pathology (68). 

Parallel to gastrointestinal and skin manifestations, where tTG-2 and 3 are involved, tTG-6 is believed to be playing a key role in the pathogenicity of nervous system manifestations of CD (69). The enzyme, here, acts in the maturation of motor function (70). Interestingly, in genomic terms, tTG-6 resides on the same chromosome (20q11–12) as tTG-2 (71) and shares a degree of functional similarity with it as well (70). Variety of mechanisms have been proposed to cause neuronal damage including cross reacting antibodies, direct toxicity and possible immune complex disease (72). The attack is specifically directed towards PC as suggested by Hadjivassiliou and colleagues (73) who demonstrated that sera from patients with GA-stained PC preferentially. 

Thus, there is a relationship between tTG-6 and neurological pathology in GA and it is argued that anti tTG-6 cross reacts with PC after blood brain barrier has been crossed (49). Furthermore, analysing sera from patients with neurological illness, Hadjivassiliou et al. (74) suggested that the presence of tTG-6 may be rightly regarded as a marker for neurological disease independent of enteric disease. On the other hand, tTG-6 is not specific for GA and may be found in multiple sclerosis as reported by a study (n=248) by Cristofanilli et al. (75) who examined the CSF samples of patients with multiple sclerosis and found high titre suggesting an independent association of tTG-6 with neuronal injury and indeed a marker of severity. Furthermore, the role of other tTGs (2 and 3) has been implicated based on cross reactivity of patient’s sera with these antigens (76). Although anti tTG-6 is suggested to serve as a biomarker for GA (50,77), it is not radially available everywhere (49) and may not be specific for GA as well (75), hence debatable in terms of its complete role in the pathogenesis (78). Further research is needed to explore the strength of independent causative role of tTG-6 in GA by developing anti tTG-6 blockers and examining them in a blinded prospective fashion. 

Anti tTG-6 are relatively sensitive and specific (77) for GA but another contending antibody, the anti-glidan antibody (AGA) has been found to have fourfold higher odds in CD with idiopathic cerebellar ataxia than controls as suggested by a systematic review (79). Additionally, Hadjivassiliou and colleagues (73) demonstrated that sera from patients with GA stained rodent PC in pain similar to the commercially available GA suggesting toxic effects on PC in relation to GA. The association of AGA with ataxia is not clear as earlier research (80,81) and the study conducted by Wong et al., (82) have contradictory findings. The reason for this discrepancy might well explain the cut-off line for AGA positivity in these studies. Hadjivassiliou at al. (83) conducted an intervention study on patients (n=21) with GA and low titres of AGA but without enteric CD and observed the effect of GFD on the improvement of GA as assessed by MR spectroscopy. They reported benefits of GFD in such patients and suggested that the level of AGA should be redefined to make it more sensitive to pick GA without enteric CD. It is also possible that yet another set of antibodies, i.e., anti-gangliodise antibodies (AGSA), are at play to have caused neurological damage as suggested by Volta et al. (84) who detected positive AGSA in 64% of patients with neuronal injury. More prospective studies are needed to analyse antibody profile in GA to reveal the exact role of those antibodies. 

The association of neurological injury with non-coeliac gluten sensitivity (NCGS), a related but pathologically different condition (85), implies an independent mechanism of neuronal injury by gluten in the absence of CD (32,86,87). Hadjivassiliou et al. (44) have also described that NCGS may also have similar neuropathic spectrum and response to GFD. The study (n=562) was retrospective and compared clinical, radiological and neurophysiological data spanning over 20 years. Likewise, Rodrigo and colleagues (88) examined groups of patients with CD, GA and NCCD and suggested that GA may well be associated with NCGD. This area thus needs further exploration to explore alternative pathways.

**Gluten-free diet and neuronal pathology**

To date, GFD is the only therapeutic option for CD (15,89) and similar observations have been recorded in relation to GA as well. This improvement is clinical as well as radiological as suggested by a study (n=117) which analysed pre- and post-GFD MR spectroscopic images and reported improvement (90). The improvement in gluten-related disorders comes with an exception that although histological changes may reverse completely in intestinal mucosa (91), in GA, gluten-free diet (GFD) may help in salvaging future neuronal loss (36,92) since an established PC loss is an irreversible phenomenon (49) and may not improve. It is, thus, important that an early diagnosis is established as suggested by a recent review paper (93). 

The adherence to GFD, however, must be strict for full therapeutic effects (94). Additionally, the use of steroids in enteric CD is rare (95-97) and reserved for refractory cases. Burke and colleagues (98) used immunoglobulin (IG) in the management of GA in a small uncontrolled study and reported improvement. This area, however, needs more research as the use of IG in ataxia with a known triggering factor may first warrant removal of the offending agent.

## Conclusion

We discussed that CD is a multisystem disease and affects patients neurologically, albeit in a small proportion of patients. There is a demand for more research, especially in establishing the exact role of immuno-pathogenesis of GA. Because of the permanent loss of associated PC, both gastroenterologists and neurologists need a degree of awareness to exclude this diagnosis before the permanent damage ensues in neuronal circuits or other systematic complications of CD emerge. The other area which needs further exploration is the antibody profile in both CD and NCGS. 

Notably, the studies presented above represent different aspects of neuropathology, yet they are different in methodology as well as the selection of patients, so it might be that the reported GA may be dependent on several factors such as malabsorption status of CD, age of diagnosis and adherence to a GFD. To correct these confounding factors and limitations of the previous studies, a multi-centre prospective study recruiting both Caucasian and non-Caucasian populations should be conducted with a control arm, relatively high power, and a long follow-up period. Such studies can hopefully demonstrate the prevalence and immunological pattern of GA in different ethnic groups not previously studied. More objective measures should be used to diagnose and monitor the progression of ataxia such as the “scale for the assessment and rating of ataxia” (99). The results can link the degree of adherence to GFD as assessed by expert dieticians.

## Conflict of interests

The authors declare that they have no conflict of interest.
